# Quality of health care with regard to detection and treatment of mental disorders in patients with coronary heart disease (MenDis-CHD): study protocol

**DOI:** 10.1186/s40359-019-0295-y

**Published:** 2019-04-08

**Authors:** Samia Peltzer, Hendrik Müller, Ursula Köstler, Katja Blaschke, Frank Schulz-Nieswandt, Frank Jessen, Christian Albus, on behalf of the CoRe-Net study group, Lena Ansmann, Peter Ihle, Ute Karbach, Ludwig Kuntz, Holger Pfaff, Christian Rietz, Nadine Scholten, Ingrid Schubert, Stephanie Stock, Julia Strupp, Raymond Voltz

**Affiliations:** 1 Department of Psychosomatics and Psychotherapy, University of Cologne, Faculty of Medicine and University Hospital Cologne, Kerpener Straße 62, 50937 Cologne, Germany; 2 Department of Psychiatry and Psychotherapy, University of Cologne, Faculty of Medicine and University Hospital Cologne, Kerpener Straße 62, 50937 Cologne, Germany; 3 Faculty of Management, Economics and Social Sciences, University of Cologne, Albertus-Magnus-Platz, 50923 Cologne, Germany; 4 PMV research group, Faculty of Medicine and University Hospital Cologne, University of Cologne, Herderstraße 52, 50931 Cologne, Germany; 5 German Center for Neurodegenerative Diseases (DZNE), Sigmund-Freud-Str. 27, 53127 Bonn, Germany

**Keywords:** Coronary heart disease, Mental disorders, Cognitive impairment, Value-based health care

## Abstract

**Background:**

Mental disorders (MD), such as depression, anxiety, and cognitive impairment, are highly prevalent in patients with coronary heart disease (CHD). Current guidelines on cardiovascular diseases recommend screening and appropriate treatment of MD; however, the degree of implementation of such recommendations in clinical practice is unknown. This study aims to analyze the quality of health care of patients with CHD and MD. Specifically, we aim to analyze (1) the quality of care, (2) trajectories of care, and (3) barriers regarding the detection and treatment of MD. Moreover, we want to identify potentials of changes in health care delivery towards more patient-centered care. The results of this study shall be the first step towards value-based care of people with CHD and comorbid mental disorders.

**Methods:**

We aim to include the following participants: adult patients with CHD (*n* = 400), their relatives (*n* = 350) and physicians (*n* = 80). A particular focus will be on the vulnerable subgroups of patients with CHD and congestive heart failure (left ventricular ejection fraction < 40%) and on the underrepresented group of women with CHD. We will apply a mixed-method approach with a quantitative and a qualitative part.

Patient-related outcomes (e.g., health-related quality of life, needs, and preferences regarding health care, reasons for non-detection, and lack of treatment of MD) will be explored in a multi-perspective approach including patients, relatives, and physicians’ perspectives. Furthermore, routine data from four statutory health insurance funds (SHI) will be analyzed regarding the frequency and treatment of MD in CHD patients.

**Discussion:**

MenDis-CHD will provide important insights into the trajectories of health care, quality of health care, barriers, patient needs and preferences as well as expectations and satisfaction with health care in patients with CHD and MD. Potential implications of MenDis-CHD are to enable health care providers to redesign care pathways concerning the treatment of mental comorbidity in patients with CHD by proposing value-based changes in health care and by understanding the barriers to and facilitators of change towards patient-centered care.

**Trials registration:**

German clinical trials register (Deutsches Register Klinischer Studien, DRKS) ieRegistration Number: DRKS00012434, date of registration: May 11th, 2017.

## Background

Coronary heart disease (CHD) is the leading cause of morbidity and mortality in Europe and the USA [1, 2]. In 2008, approximately 17 million people died worldwide from CHD, and 10% of the burden of disease worldwide is caused by CHD [3]. Due to advances in the acute treatment of CHD, short-term mortality has decreased, but morbidity on a population level has increased [1, 2, 4]. In recent years, gender aspects concerning CHD received increasing attention. Men have a twofold higher CHD prevalence (12.3%) than women (6.4%) [4, 5]. However, there is a stronger increase in incidence in older women compared to men [4]. CHD-related mortality in women also appears to be slightly higher [5]. Several reasons have been discussed, including the older age of women at the acute cardiovascular event, but also lower detection rates and less treatment of cardiac symptoms [5, 6]. Also, mental disorders (MD) have been found to be highly prevalent among patients with CHD, particularly in women [7]. Especially depression, anxiety disorders, cognitive impairments [8] and other MDs affect approximately 50% of all CHD patients [7, 9, 10].

MD – whether they are pre-existing conditions or consequences of CHD – act as particularly strong barriers to treatment adherence and impede efforts to lifestyle change [1], resulting in an approximately two-fold greater morbidity and mortality risk in CHD [1, 7, 9, 11, 12].

In detail, several mechanisms are discussed: first, unhealthy lifestyle (e.g., smoking, high alcohol consumption) is more prevalent in patients with CHD and MD. Second, CHD-patients with MD are more resistant to behavior change and have low medication adherence. Third, psychobiological mechanisms are found, which increased risk for CHD, even if the ‘classical’ risk factors are controlled for (e.g., alterations in autonomic functions, in the hypothalamic-pituitary axis and in inflammatory markers [13]). The prevalence of depressive symptoms in CHD patients is approximately 31% in women and 20% in men. Anxious symptoms occurred approximately in 39% of the women and in 22% of the men 1.4 years after hospitalization for CHD [14]. Consequently, recent, national and international guidelines on primary and secondary prevention of CHD recommend routine screening for MD and adequate treatment for at-risk patients [1, 10, 15].

Given that adherence to CHD guidelines in physicians is generally low, even when these guidelines predominantly comprise somatic recommendations [16], it is unlikely that general practitioners and cardiologists regularly screen for MD due to typical restraints such as a lack of time and low reimbursement of verbal interventions [12, 17]. However, a systematic review in 2013 showed that depression screening is only beneficial for identifying depressed patients that were not already diagnosed or treated for depression. Also, the accuracy of screening tools seems to be exaggerated by the inclusion of already diagnosed patients and the selective reporting of results from cut-off scores. Following this review, a wide range of routine screening would entail high costs and increase the number of patients using antidepressants. Hence, it is important to not only look at the screening for MD and cognitive impairements but also to look at diagnostic and treatment following the screening [18]. Overall, the status of quality of care in CHD patients with comorbid mental disorders is unknown. Moreover, it is unclear what the trajectories of care of patients with CHD and cognitive impairement are.

In addition, no data on personal preferences, needs, and expectations of patients with CHD and their relatives regarding the detection and treatment of MD have been published. Thus, there is an urgent need to explore the current state of care in patients with CHD and MD to identify relevant factors that are preventing improved care in health care systems.

## Methods

### Aims

The study examines [1] the current quality of health care regarding to the detection and treatment of MD in patients with CHD, [2] the experiences of physicians in treating their patients according to guideline, [3] needs and preferences of the patients and [4] possible barriers for the implementation of guideline-based diagnostic and treatment.

Further, routine data from health insurance funds (SHI), collected continually for reimbursement and stored for several years by health insurers, will be allocated [19]. The SHI-data will be analyzed concerning frequency of CHD as well as sex and age distribution, use of the resource, costs, and trajectories of care (e.g., diagnosis of MD, psychotropic medication, psychotherapy, hospital stay, sick leave certificates, and early retirement).

The findings of the MenDis-CHD will contribute to an overview of the current state of health care in CHD with the aim of improving and modifying care delivery, so that appropriate interventions ensure value-based health care.

### Theoretical framework and research platform

MenDis-CHD is located in Cologne, Germany and is one of three current projects of the ‘Cologne Care Research and Development Network’ (CoRe-Net). Core-Net is funded by the Federal Ministry of Education and Research (BMBF), and the MenDis-CHD study protocol was subject to peer review by the funding body before approval. The value-based health care concept by M.E. Porter [20–22] forms the framework of CoRe-Net, which has the aim to create a data-driven learning environment employing research and practice to improve health and social care organizations by transforming them into a system that develops and delivers care with greater value. We define value-based health care as cost-consciously redesigning care processes and structures according to the needs of patients, which includes the two dimensions of patient-related and economic aspects. For further details about CoRe-Net see [23].

### Participants of MenDis-CHD

Overall, we aim at recruiting a total number of *N* = 830 participants. The total sample includes subsamples of patients, relatives, and physicians. Since Jan 15, 2018, the MenDis-CHD study is in the recruitment phase.

#### Patients

We intend to enroll adult patients (*n* = 400; 50% women) with angiographically documented CHD treated for stable angina pectoris, acute coronary syndromes, percutaneous coronary intervention, or bypass surgery. Participants must be able to give informed consent and have sufficient German language skills. Exclusion criteria are severe or instable physical or mental conditions (e.g., severe illnesses such as cancer, acute suicidal ideation, delirium, and moderate to severe dementia). Since we are conducting a descriptive and exploratory study, but not a confirmatory one, we did not performed a sample size calculation based on a power calculation. Rather the focus of MenDis-CHD is on vulnerable subgroups. These vulnerable subgroups comprise (1) older female CHD-patients which were underrepresented in past studies, (2) CHD-patients with comorbid mental disorders and/or mild cognitive impairment (expected prevalence of MD and MCI is 30–50%), and (3) CHD-patients with congestive heart failure (expected prevalence of 30% with a left ventricular ejection fraction of < 40% [24]). To obtain a realistic estimate of the health care situation, patients will be recruited in cardiology departments of hospitals, cardiology practices, and rehabilitation clinics.

Thus, the rational for the intended sample size are this estimated frequency of the gender distribution, mental and cognitive comorbidity and patients with congestive heart failure as well as the goal of recruiting in different sectoral areas (hospitals, rehabilitation clinics, and cardiology practices). By this rationale, we aim at a sufficiently large sample sizes to perform the statistical comparison in these vulnerable subgroups. In sum, the recruitment of a total *N* of 400 patients is planned. Thus, we aim to recruit *n* = 200 women, *n* = 130–200 patients with MD or MCI and *n* = 120 with congestive heart failure.

All 400 patients (with and without MD) will take part in the quantitative study. *N* = 20 participants of the patient sample will be invited to join qualitative interviews.

#### Relatives

We aim at recruiting a total number of *n* = 350 relatives of the CHD patients for the quantitative survey. Of these, *n* = 20 will be asked to participate in qualitative interviews. The group of relatives is defined as every close person living in the household of the patients. Inclusion criteria for relatives are: 18 years of age or older, able to give informed consent and sufficient German language skills. Exclusion criteria are severe or instable physical or mental conditions.

#### Physicians

A total number of *n* = 80 physicians (general practitioners (GPs), cardiologists, physicians at rehabilitation clinics, and psychotherapists) will be tried to be recruited for a quantitative assessment. *N* = 20 physicians of this sample will also participate in qualitative interviews. Moreover, *n* = 40 physicians will be randomly selected to participate in focus groups. Four focus groups, each consisting of ten participants will be conducted, including following specialties named above.

**Fig. 1:**
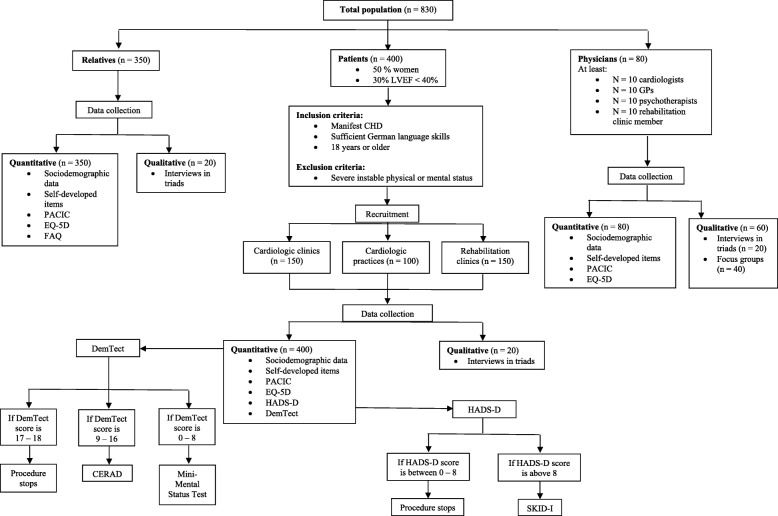
Flowchart of the study population divided into three sub populations with the detailed procedure. CERAD: Consortium to Establish a Registry for Alzheimer’s disease; CHD: Coronary Heart Disease; DemTect: Demenz-Detection-Test; EQ-5D: EURO-Quality of Life 5D; FAQ: Functional Activities Questionnaire; GPs: General Practitioner; HADS-D: Hospital Anxiety and Depression Scale; LVEF: Left Ventricular Ejection Fraction; PACIC: Patient Assessment of Care for Chronic Conditions; SKID-I: Structured Clinical Interview for DSM-IV

Please refer to Fig. 1 for details on the recruitment procedure and sub-samples.

#### Study population of SHI data

SHI data of patients who are inhabitants of Cologne continually insured between 2011 and 2012 and continually insured or deceased in 2013 through 2015 in one of the four participating health insurance companies - (estimated about 270,000 persons) will be provided.

### Assessments

The design of MenDis-CHD is a monocentric cross-sectional mixed methods approach [25] comprising quantitative (primary and SHI data as secondary data) and qualitative research (interviews and focus groups).

#### Quantitative studies

##### Disease severity

We assess disease severity of CHD by ascertaining the reason of admission to hospitals, cardiology practices, and rehabilitation clinics, the actual therapy, secondary diagnoses, cardiovascular risk factors, left ventricular ejection fraction, NYHA (New York Heart Association) and actual medication. If applicable, we assess severity of cardiac events (e.g., heart attack), congestive heart failure, bypass-surgery, percutaneous coronary intervention, cardiac valve surgery, echocardiography, cardiac catheterization.

##### Clinical questionnaires

The *Hospital Anxiety and Depression Scale* (HADS; [26, 27]) will be used as a screening tool for MD. The HADS is a self-report questionnaire to assess symptoms of depression and anxiety. It contains 14 items, which can be rated on a 4-point Likert-scale. Depression and anxiety sub-scales can be extracted. An anxiety/depression score between 0 and seven is considered ‘negative,’ between eight and ten ‘sub-syndromal positive’ and between 11 and 21 ‘positive’ for depression and/or anxiety. The reliability for the anxiety scale is α = .80, for the depression scale α = .81. The retest-reliability within 2 weeks is α = .80 for the anxiety scale and α = .83 for the depression scale. The construct validity is between α = .06–.08. Cronbach’s Alpha of the HADS is 0.80, and the retest is indicated as α = .80 [28].

The sensitivity and specificity for case detection are around .80 for both scales [28]. HADS-screening will be defined as ‘positive’ if the score is greater or equal to eight. In this case, the *Structured Clinical Interview for DSM-IV* (SKID-I; [29]) will be conducted. The SKID-I is a semi-structured interview to assess psychiatric symptoms and disorders as defined in the DSM IV (*Diagnostic and Statistical Manual of Mental Disorders*; [30]). Lifetime and current diagnosis are assessed for the following disorders: [1] affective disorders, psychotic disorders, disorders through psychotropic substances, anxiety disorders, somatoform disorders, eating disorders and adjustment disorder. The test-retest reliability relevant for this study are within the range of α = .61–.76 for unipolar affective disorders and α = .65–.63 for anxiety disorders [31]. The sensitivity for affective disorders is .53 and for major depression .84. The specificity for affective disorders is .97 and for major depression .91 [32].

The *Demenz-Detection-Test* (DemTect; [33, 34]) will be used as a screening instrument to assess cognitive impairments. The DemTect contains five tests (1) a word list with immediate recall, (2) a number transcoding task, (3) a word fluency task, (4) a digit span reverse, and (5) a delayed recall of the word list. The total score of the DemTect gives an estimate whether the cognitive performance of the participant is normal for age (13–18 points), or whether mild cognitive impairment (MCI, 9–12 points) or dementia (8 points or below) should be suspected. In this study, we defined a DemTect score between 9 and 16 points as potentially indicative of cognitive impairments and thus will be regarded as ‘positive’. In case of a positive screening with the DemTect, the *Consortium to Establish a Registry for Alzheimer’s Disease* test battery (CERAD-Plus [35]) will be applied. If the DemTect score equals eight or is lower, the *Mini-Mental State Examination* (MMSE, 36) to detect significant cognitive deficits will be conducted. The test-retest reliability and the construct validity is above α = .80. The sensitivity is .97 and specificity is .93 [34]. The CERAD-Plus is a neuropsychologic test battery for the diagnostic of dementia [35]. It contains ten sub tests, which assess cognitive performance in different cognitive domains: (1) verbal fluency, (2) Boston naming test, (3) Mini-Mental Status Examination, (4) word list learning, (5) constructional praxis, (6) word list delayed recall, (7) word list recognition and discriminability, (8) constructional praxis delayed recall, (9) Trail Making Test A and B, and (10) phonematic fluency. The raw data will be z-standardized and adjusted for age, gender and education. If the z-score is between − 1.0 and − 1.5 SD, the cognitive performance is considered mildly impaired and below − 1.5 as severe impaired.

The *Mini-Mental Status Examination* [36] is a screening instrument to assess signs of dementia. The maximum is 30 points. The scores are interpreted as follows: 30–27 point: no evidence of cognitive impairment, 26–20 points: indicative of mild dementia, 19–10 points: indicative of moderate dementia, nine points and below: indicative of severe dementia.

The test-retest reliability is α = .89, and the construct validity is comparable to the construct validity of the DemTect [37]. The sensitivity is .88, and the specificity is .86 [38].

To estimate functional abilities in cognitively impaired patients, all participating relatives are asked to fill out the *Functional Activities Questionnaire* (FAQ; [39]). The FAQ contains ten items and rates the patient’s ability to perform daily activities. Participants rate each item on a 4-point Likert-scale ranging from 0 = ‘normal’ to 3 = ‘dependent’. The scores are added to a sum score of 30. If the score equals nine or is higher an impaired function in daily activities is indicated. The construct validity is α = .847, the sensitivity is α = .803 and the specificity is α = .870 [40].

##### Health care questionnaires

Quantitative research in CHD patients, relatives and physicians will assess patients’ trajectories and quality of care, barriers to guideline-based care, health care preferences, quality of life, the presence of MD, disease severity and provided health care.

The three versions of the health care questionnaire comprise in total 158 items for patients, 147 items for relatives, and 76 items for physicians. Questions are for example: ‘Do you communicate with your physician (e.g., GP or cardiologist) about mental health issues?’

Furthermore, specific interventions and quality of health care in the enrolled patients are assessed by the *Patient Assessment of Care for Chronic Conditions* (PACIC; [41]). The PACIC contains 26 items, which are each rated on a 5-point Likert-scale ranging from 1 = ‘almost never’ to 5 = ‘almost always’. The higher the PACIC score, the better the patient-centeredness in health care from the patient’s point of view. Cronbach’s Alpha of the PACIC is 0.93, and the retest is indicated as α = .58 [41].

The *EURO-Quality of Life 5D* questionnaire (EQ-5D; [42]) measures the health-related quality of life in five dimensions (mobility, self-care, usual activities, pain/discomfort, anxiety/depression) with three items per dimension. The items are rated on a 3-point Likert scale from 1 = ‘no problems’ to 3 = ‘extreme problems.’ The higher the score, the worse is the health-related quality of life. The test-retest reliability for the five sub scales are α = .69, α = .77, α = .64, α = .48, and α = .61, respectively. All five sub scales are summed up in the life-quality index with a test-retest reliability of α = .75. The visual analog scale is a 20 cm vertical visual scale with endpoints ‘the best health condition you can imagine’ and ‘the worst health condition you can imagine.’ The visual analog scale can be used as a quantitative individual measure of health with a test-retest reliability of α = 92 [43].

##### Questionnaires for physicians

All professional health care providers (*n* = 80) will be quantitatively surveyed in cooperation with the OrgValue project of CoRe-Net. OrgValue (Organization & Value) focusses on patient-centeredness and on economic aspects (resources, costs, payment). OrgValue analyzes the health care organizations involved in the care of patients studied in MenDis-CHD using a structured questionnaire to assess their knowledge, attitudes, and experiences concerning patient needs and preferences and barriers associated with MD detection and treatment.

##### Statutory health insurance data (SHI)

Secondary claims data will be provided by four major health insurances of the state of North Rhine-Westphalia in Germany, from 2011 to 2015, for insuree living in Cologne. This SHI data is part of the CoRe-Net-database and will be used for this project (https://www.core-net.uni-koeln.de/index.php/de/core-net-datenbank/). Besides master data (e.g., age, sex, insurance status and period of insurance) information concerning the use of all sectors of care (inpatient and outpatient care, drug prescription, benefits in kind, long-term care) will be available and connectable by a non-identifiable study number. In detail, ICD-10 coded diagnoses from out- and inpatient care, medical services according to the EBM-Code (German physician fee manual), hospital stays with length of stay and OPS-procedures (Operations and Procedures Key), drug prescription with pharmaceutical registration number and linkage to ATC-Code (Anatomical Therapeutic Chemical Classification System) and DDD (Defined Daily Dose Classification), as well as information concerning inability to work (diagnosis, duration) and utilization of long-term care will be provided. For all services, provided SHI cost data will be available. Spatial data (e.g., INKAR: Indicators and maps for area- and town development) could be added.

In a first step, quality and plausibility checks of the SHI-data are performed. In a second step, patients with CHD and MD are identified by their diagnoses, which will be internally validated [44]. Inclusion criteria for CHD patients will be a hospital discharge diagnosis of CHD (ICD 10-GM code I20- I25), for chronic heart failure patients (ICD 10-GM code I11.0, I13.0, I13.2, I50), an outpatient diagnosis in at least two quarters of a or a hospital discharge diagnosis and for MD-patients (ICD 10 GM-code F00-F99, and more specific: F06.7, F32, F33) in one quarters of year or a respective hospital diagnosis. We will apply a cross-sectional design for basic epidemiological data (e.g., frequency of CHD, CHD, and MD, psychotherapy, mortality) and a cohort design for the analysis of care trajectories starting in 2013, allowing a pre-observation period and a follow up of at least 2 years each.

These SHI-data will enrich the empirical part of the study [19] [45] as it allows to compare characteristics of general CHD-population with MD identified in the data to those included into the study and to determine the possible selection bias.

#### Qualitative studies

The qualitative module is designed to build on the questions of the quantitative module. With regards to those questions, the research design provides for data collection by interviews based on a predefined guideline. Guiding questions are for example: ‘How are the patients experiencing health care?’, ‘In how far is the practitioners’ advice complied with and to what extent can patients cope with their condition?’ The coping of the relatives with regard to the patients disorder will be also assessed.

##### Focus groups

Regarding the focus groups, the interpretations of the involved medical professionals will be analyzed [46]. It is of interest whether medical professionals are oriented towards national and international guidelines on primary and secondary prevention of CHD, especially the recommended routine screening for MD [1, 9]. The chosen procedure serves to inform the following face-to-face interviews.

##### Interviews in triades

Secondly, we plan to perform 60 face-to-face interviews – selected as triads, each of those with a patient, his or her relatives, and the responsible practitioner.

**Fig. 2:**
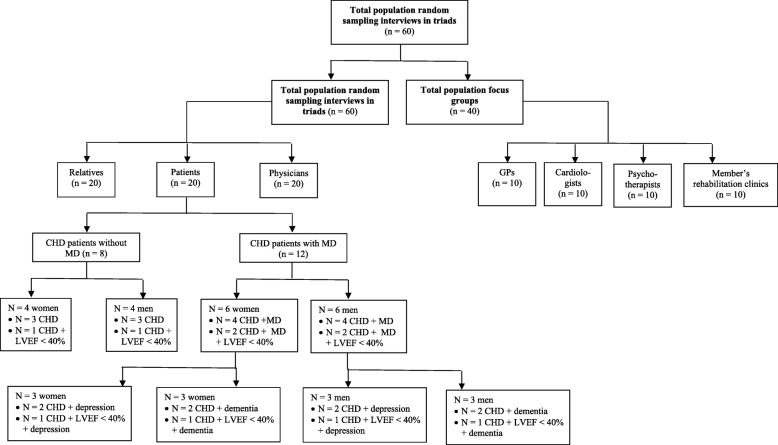
Flowchart of samplings for interviews and focus groups. CHD: Coronary Heart Disease; GPs: General Practitioner; LVEF: Left Ventricular Ejection Fraction; MD: Mental Disorders

Please refer to Fig. 2 for details on the recruitment procedure and subsamples of the qualitative part of the study.

### Procedures

The University Hospital of Cologne (Department of Psychosomatics and Psychotherapy and Department of Psychiatry and Psychotherapy) is the only recruiting site. However, participants will be recruited in cardiology departments (University Hospital Cologne, Department of Internal medicine, Cardiology, Pneumology and Internal Intensive Care Medicine; Cologne-Merheim, clinics of the city of Cologne, Department of Cardiology, Rhythmology and Internal Intensive Care Medicine), patients from two cardiologic rehabilitation clinics (Clinic Roderbirken - Rehabilitation Centre for Heart and Circulatory Diseases; AmKaRe Cologne: out-patient cardiological rehabilitation centre) and from three cardiologic practices (Practice for Internal Medicine, Cardiology, Pneumology, Cologne, Wiener Platz 1; Practice for Cardiology Cologne, Josef-Haubrich-Hof 5; Practice for Cardiology Cologne, Wehrtmannstr. 1b). All patients screened for eligibility will be documented in a screening log. Patients who fulfill all inclusion and no exclusion criteria and provide written informed consent will be handed out the quantitative questionnaires and will be asked to participate in the qualitative interviews. If this screening with the HADS and/or the DemTect is positive, a second appointment will be arranged to perform the SKID-I and/or the CERADPlus. All participating researchers (SP and HM) were trained in applying the SKID-I and CERADPlus and are experienced in conducting the interviews with patients and research participants. The subsample of 20 participants, 20 relatives, and 20 physicians will be randomly contacted for a qualitative interview at a third time point. Furthermore, focus groups with 40 physicians will be randomly contacted.

### Outcomes

#### Quantitative

The research questions of the quantitative module are: (1) Are actions for MD detection and treatment taken? Are these actions consistent with national and international guidelines on primary and secondary prevention of CHD? (2) What are the experiences of GPs and cardiologists who treat their CHD patients according to the guidelines? For those GPs and cardiologists who do not adhere to the guidelines, what are the underlying reasons? (3) Do the assessment and treatment of MD correspond to CHD patients´ needs and preferences? (4) What kind of treatment is offered for CHD patients diagnosed with MD? Are patients supported in finding the appropriate treatment? For what reasons do patients reject offered services or are not satisfied with them? (5) What are the barriers for a correct implementation of guideline-based diagnostic and treatment? What changes do the GPs and cardiologists suggest?

#### Qualitative

The qualitative module aims to analyze profoundly the patients’ needs, preferences, attitudes, and barriers regarding value-based care of CHD patients with comorbid MD. The overall research question of the qualitative module focuses on the trajectories of care and quality care of the CHD-patients, relatives, and physicians. In how far are their expectations met and which barriers are they facing?

Overall, outcomes of the patients’ data are: (1) prevalence of MD, (2) types of diagnostic procedures and treatment received, (3) quality of life, (4) satisfaction and short comings with respect to trajectories and quality of health care, and (5) expectations and needs with regard to health care (patient preferences). The outcomes will be analyzed separately for age groups and gender.

Main outcomes for the relatives’ data are: (1) frequency of contact with the health care system and provided care, (2) caregivers’ burden, (3) quality of life, (4) satisfaction with respect to trajectories and quality of health care, (5) patterns of perception about value, and (6) expectations and needs with regard to health care (preferences of relatives).

Main outcomes of the health care professionals’ are: (1) knowledge, attitudes, and experiences concerning guideline recommendations, (2) personal views and experiences regarding MD detection, treatment, and value for the patient, and (3) limitations and barriers in the health care system. Further, outcomes of the focus groups of the health care professionals are about obstacles about guideline adherence, diagnosis and treatment of MD in CHD.

In the SHI data the outcomes of interest are prevalence and incidence of MD diagnosis in CHD patients, documented diagnostics, and treatments (e.g., prevalence of psychotropic medication and/or psychotherapy), documented costs according to sectors of care, as well as treatment persistence, frequency and duration of hospital stay, sick leave certificates, early retirement and death.

#### SHI data

We will provide frequency measures for patients with CHD in general and for those with (1) MD present before the hospital stay and (2) MD documented for the first time, i.e. no hint for MD by diagnosis or drug prescription in the interval of one respective two years before index stay (the MD group will be of main interest). This respective hospital stay serves as an index period, which allows the description of pre and post use of health care services and care trajectories. Besides estimating prevalence for CHD and CHD with comorbidity like MD, the data allows to assess by whom and how patients are treated (specialty of physician group, diagnostic procedures, non-medical therapy, drug prescribing, further hospital stays) and how patients comply with the treatment (adherence to medication regime according to the PDC methodology expressing the percentage of days covered with medication [47, 48]). Absent days at work, early retirement and medical costs will also be analyzed. Predictors for MD treatment related to the information available in claims data will be assessed.

## Analysis

### Statistical analysis

Analysis of the quantitative data will include descriptive statistics, exploratory analysis (e.g., regression analysis), sub-group analysis and multivariate analyses to identify predictors of features of quality of health care. Therefore, statistical analyses will be exploratory and not confirmatory. Descriptive and analytic statistics will also be used for the analysis of the SHI data [49].

### Content analysis

Qualitative data will be subject to content analysis. By using methods of qualitative social research, the study intends to demonstrate effects and relations using an exploratory approach and build on the interpretative method of Rosenthal [50].

## Discussion

Data will be generated from multiple sources, including claims data, surveys, interviews and focus groups of professionals, patients, and relatives. Thus, our study design adopts a multi-perspective approach, which combines patients, their relatives and physicians views with in-depth analysis of trajectories, quality of health care, needs and preferences by quantitatively and qualitatively methods. Besides, we are able to gain insight into the general treatment of a non-selective population with CHD and MD by using SHI data.

The value-based concept by Porter [20–22] forms the analytical framework for our project. Consequently, our vision is to create value for the patient paying attention to both quality of health care and costs. MenDis-CHD will provide essential insights into the trajectories of health care, quality of health care, barriers, patient needs and preferences as well as expectations and satisfaction with health care in patients with CHD and MD.

The multi-method and multi-perspective approach of MenDis-CHD will provide data-driven analysis tools to enable care providers to redesign care pathways by proposing value-based changes in care and to understand the barriers to and facilitators of change towards patient-centered care. Restructuring complex care for vulnerable CHD patients is very much needed since there is evidence that these patients are underserved and often get lost in the transition between multi-professional and multi-institutional care providers [51, 52]. This generates unnecessary costs, ill health and thus low value for the patient.

MenDis-CHD has already begun to perform workshops inviting our scientific and practice partners, stakeholders, caregivers, patient and relative representatives to develop specific deliveries and methods for value-based health and social care in highly vulnerable patients with CHD and MD. Moreover, we have built up a representative network of recruitment centers. However, practice will have to show how well we succeed in recruiting patients against the backdrop of a significant reduction in inpatient stays. As already noted, women represent a vulnerable subgroup underrepresented in previous studies. Thus, recruitment will have to additionally show how demanding it is to achieve the targeted sample sizes of female patients.

The patient-centered products could be (a) an improved model of value-based health care for patients with CHD and MD (new standardized pathway with safe transitions), (b) a generalizable approach for transforming this model to other somatic patient groups with mental comorbidities and (c) gender-specific prompt sheets for patients.

Relatives-centered products may be (a) training units for relatives to enhance their ability to co-manage the process of comorbidity health care and (b) gender-specific prompt sheets for relatives. Professional-centered products could be (a) recommendations to improve professional cardiological guidelines and (b) trajectory-related directories as a coordination tool. The organization-centered products are self-analysis tools to raise awareness about mental comorbidities. Ultimately, we aim to improve the health care of people with somatic and mental disorders.

## Abbreviations

ATC: Anatomical Therapeutic Chemical Classification System
BMBF: Bundesministerium für Bildung und Forschung (English: Federal Ministry of Education and Research)
CERAD: Consortium to Establish a Registry for Alzheimer’s disease
CHD: Coronary heart disease
CoRe-Net: Cologne Care Research and Development Network
DDD: Defined Daily Dose Classification
DemTect: Demenz-Detection-Test
DSM IV: Diagnostic and Statistical Manual of Mental Disorders
EQ-5D: EURO-Quality of Life 5D
FAQ: Functional Activities Questionnaire
FHS: Faculty of Human Sciences
FM: Faculty of Medicine
FMESS: Faculty of Management, Economics and Social Sciences
GP: General Practitioner
HADS-D: Hospital Anxiety and Depression Scale
ICD-10: International Statistical Classification of Diseases and Related Health Problems, Version 10
IMVR: Institut für Medizinsoziologie, Versorgungsforschung und Rehabilitationswissenschaft (English: Health Services Research and Rehabilitation Science)
INKAR: Indikatoren und Karten zur Raum- und Stadtentwicklung (English: Indicators and maps for area- and town development)
LVEF: Left Ventricular Ejection Fraction
LYOL-C: Last Year Of Life in Cologne
MCI: Mild Cognitive Impairment
MD: Mental Disorders
MenDis-CHD: Mental Disorders in coronary heart disease
MMSE: Mini Mental State Examination
NYHA: New York Heart Association
OPS: Operations and Procedures Key
OrgValue: Organization & Value
PACIC: Patient Assessment of Care for Chronic Conditions
PDC: Percentage of days covered (measure for adherence)
SHI: Statutory Health Insurance Data
SKID-I: Structured Clinical Interview for DSM-IV
UHC: University Hospital Cologne
UoC: University of Cologne
ZVFK: Zentrum für Versorgungsforschung Köln (English: Center for Health Services Research Cologne)
